# Supplementary material for: Mapping of the podocin proximity-dependent proteome reveals novel components of the kidney podocyte foot process
Source: Front Cell Dev Biol. 2023 May 30;11:1195037. doi: 10.3389/fcell.2023.1195037 (PMC10262054; doi:10.3389/fcell.2023.1195037)
Supplement: Supplementary file 3 [file DataSheet1.pdf]

Mass Spec #1		Mass Spec #2		Mass Spec # 3		
Control Males	*Nphs2*$^{BioID2/+}$ Males	Control Males	*Nphs2*$^{BioID2/+}$ Males	Control Females	*Nphs2* $^{BioID2/+}$ Females	
Sample # 1 = 3 males	Sample # 1 = 3 males	Sample # 1 = 3 males	Sample # 1 = 3 males	Sample # 1 = 3 females	Sample # 1 = 3 females	
Sample # 2 = 3 males	Sample # 2 = 3 males	Sample # 2 = 3 males	Sample # 2 = 3 males	Sample # 2 = 3 females	Sample # 2 = 3 females	
Sample # 3 = 3 males	Sample # 3 = 3 males	Sample # 3 = 3 males	Sample # 3 = 3 males	Sample # 3 = 3 females	Sample # 3 = 3 females	
n = 9	n = 9	n = 9	n = 9	n = 9	n = 9	**Total Number of Animals = 54**
n = 18	n = 18	n = 18	n = 18	n = 18	n = 18	**Total Number of Kidneys = 108**

Supplemental Table 1. Animals utilized for mass spectrometry (MS) analysis. Table of mice and kidney samples utilized for MS. Each MS sample had the kidney cortex isolated from 3 animals for a total of 6 kidney cortices per sample. Each MS submission was 3 samples per condition, for a total of 9 animals or 18 kidney cortices per condition, with two conditions *i.e.* control and *Nphs2*$^{BioID2/+}$. The control mice were wild type C57BL/6J littermates. MS analyses #1 and #2 were run on 8-10-week-old male mice with MS analysis #3 being performed on 8-10-week-old female mice. Three MS analyses were run in total, culminating in 54 animals and 108 kidney cortices utilized in this investigation.

Gene Symbol	Majority Protein IDs	Protein Names	Razor + Unique Peptides	Log_2 Fold Change	*p* - Value
Nphs2	Q91X05	Podocin	12	10.63	0.00003
Tjp1	P39447	Tight junction protein ZO-1	85	7.40	0.001
Magi2	Q9WVQ1	Membrane-associated guanylate kinase, WW and PDZ domain-containing protein 2	33	7.37	0.001
Kirrel	Q80W68	Kin of IRRE-like protein 1	20	7.06	0.001
Pard3b	Q9CSB4	Partitioning defective 3 homolog B	32	5.34	0.00002
Ddn	Q80TS7	Dendrin	12	5.33	0.00003
Ildr2	B5TVM2	Immunoglobulin-like domain-containing receptor 2	11	4.51	0.009
Cttn	Q60598	Src substrate cortactin	15	4.01	0.0003
Nebl	Q9DC07	LIM zinc-binding domain-containing Nebulette	11	3.65	0.003
Tjp2	Q9Z0U1	Tight junction protein ZO-2	31	3.63	0.021
S100g	P97816	Protein S100-G	4	3.06	0.034
Fnbp1l	Q8K012	Formin-binding protein 1-like	7	2.92	0.010
Capza2	P47754	F-actin-capping protein subunit alpha-2	8	2.82	0.014
Atp6v1e1	P50518	V-type proton ATPase subunit E 1	9	2.30	0.039
Canx	P35564	Calnexin	9	2.26	0.042
Myzap	Q3UIJ9	Myocardial zonula adherens protein	7	2.09	0.0001
Kpnb1	P70168	Importin subunit beta-1	2	1.96	0.041
Dars	Q922B2	Aspartate--tRNA ligase, cytoplasmic	7	1.88	0.029
Cndp2	Q9D1A2	Cytosolic non-specific dipeptidase	6	1.83	0.041
Pls3	Q99K51	Plastin-3	5	1.76	0.029
Sult1d1	Q3UZZ6	Sulfotransferase 1 family member D1	5	1.73	0.038
Uqcr10	Q8R1I1	Cytochrome b-c1 complex subunit 9	3	1.67	0.048
Uba52	P62984; P62983; P0CG49; P0CG50	Ubiquitin-60S ribosomal protein L40	5	1.60	0.041
Sec23a	Q01405	Protein transport protein Sec23A	7	1.59	0.030
Cisd2	Q9CQB5	CDGSH iron-sulfur domain-containing protein 2	2	1.46	0.026
Naca	Q60817;P70670	Nascent polypeptide-associated complex subunit alpha, muscle-specific form	2	1.46	0.049
Ca15	Q99N23	Carbonic anhydrase 15	3	1.44	0.050
Qars	Q8BML9	Glutaminyl-TRNA Synthetase 1	2	1.40	0.039
Rpl35	Q6ZWV7	60S ribosomal protein L35	4	1.37	0.038
Agk	Q9ESW4	Acylglycerol kinase, mitochondrial	5	1.37	0.039
Scp2	P32020	Non-specific lipid-transfer protein	13	1.36	0.026
Aimp1	P31230	Endothelial monocyte-activating polypeptide 2	2	1.26	0.046
Dhrs7b	Q99J47	Dehydrogenase/reductase SDR family member 7B	5	1.24	0.024
Fabp4	P04117	Fatty acid-binding protein, adipocyte	2	1.17	0.012
Mpv17	P19258	Protein Mpv17	2	1.15	0.007
Sf3b1	Q99NB9	Splicing factor 3B subunit 1	4	1.10	0.039
Dkc1	Q9ESX5	H/ACA ribonucleoprotein complex subunit 4	4	1.06	0.003
Por	P37040	NADPH--cytochrome P450 reductase	11	1.05	0.031
Synpo	Q8CC35	Synaptopodin	6	1.02	0.054
Acox1	Q9R0H0	Peroxisomal acyl-coenzyme A oxidase 1	24	1.01	0.015
Cisd1	Q91WS0	CDGSH iron-sulfur domain-containing protein 1	6	0.99	0.036

Supplemental Table 2. Proteomic profiling of cohort 1, 8–10-week-old *Nphs2*$^{BioID2/+}$ male mice, identifies 41 enriched podocyte foot process proteins. MS analysis of biotin administered male *Nphs2*$^{BioID2/+}$ mice identifies podocin (Nphs2), Tjp1, Magi2, Kirrel, and Pard3b as the top 5 proteins, respectively. Table lists 41 significant proteins ($p \leq 0.05$) at a log_2 cut off 1.0 with their respective *p*-values in the far-right column. Podocin was identified as the top hit and is documented to oligomerize with itself supporting the efficacy of the model. All proteins were identified with at least 2 razor unique peptide sequences *i.e.*, two peptide sequences aligned only to that protein.

Gene Symbol	Majority Protein IDs	Protein Names	Razor + Unique Peptides	Log_2 Fold Change	*p* - Value
Kirrel	Q80W68	Kin of IRRE-like protein 1+D3:D62	15	10.19	0.001
Tjp1	P39447	Tight junction protein ZO-1	85	9.88	0.003
Pard3b	Q9CSB4	Partitioning defective 3 homolog B	26	9.62	0.016
Ddn	Q80TS7	Dendrin	11	8.99	0.005
Magi2	Q9WVQ1	Membrane-associated guanylate kinase, WW and PDZ domain-containing protein 2	24	8.61	0.011
Nebl	Q9DC07	LIM zinc-binding domain-containing Nebulette	7	7.75	0.021
Ildr2	B5TVM2	Immunoglobulin-like domain-containing receptor 2	9	7.42	0.010
Cttn	Q60598	Src substrate cortactin	14	7.08	0.020
Nphs2	Q91X05	Podocin	10	7.05	0.023
Fnbp1l	Q8K012	Formin-binding protein 1-like	5	6.65	0.008
Magi1	Q6RHR9	Membrane-associated guanylate kinase, WW and PDZ domain-containing protein 1	6	5.89	0.002
Tjp2	Q9Z0U1	Tight junction protein ZO-2	30	5.78	0.006
Myzap	Q3UIJ9	Myocardial zonula adherens protein	4	4.80	0.018
Synpo	Q8CC35	Synaptopodin	4	4.79	0.043
Khsrp	Q3U0V1	Far upstream element-binding protein 2	5	4.69	0.014
Mapre1	Q61166	Microtubule-associated protein RP/EB family member 1	4	4.56	0.019
Yes1	Q04736	Tyrosine-protein kinase Yes	8	4.34	0.015
Tmem65	Q4VAE3	Transmembrane protein 65	4	4.25	0.030
Cd2ap	Q9JLQ0	CD2-associated protein	5	4.15	0.019
Ap1g1	P22892	AP-1 complex subunit gamma-1	6	3.85	0.018
Ewsr1	Q61545	RNA-binding protein EWS	2	3.83	0.038
Oxsm	Q9D404	3-oxoacyl-[acyl-carrier-protein] synthase, mitochondrial	4	3.77	0.033
Erlin2	Q8BFZ9	Erlin-2	6	3.44	0.008
Epb41l5	Q8BGS1	Band 4.1-like protein 5	9	3.13	0.003
Hsd3b4	Q61767	3 beta-hydroxysteroid dehydrogenase type 4	9	2.99	0.028
Dhtkd1	A2ATU0	Probable 2-oxoglutarate dehydrogenase E1 component DHKTD1, mitochondrial	7	2.93	0.017
Taco1	Q8K0Z7	Translational activator of cytochrome c oxidase 1	2	2.64	0.045
Aif1l	Q9EQX4	Allograft inflammatory factor 1-like	3	2.31	0.001
Gstt2	Q61133	Glutathione S-transferase theta-2	4	2.24	0.002
Sorbs1	Q62417	Sorbin and SH3 domain-containing protein 1	18	2.21	0.036
Tns2	Q8CGB6	Tensin-2	12	2.20	0.007
Farp1	F8VPU2	FERM, RhoGEF and pleckstrin domain-containing protein 1	7	2.13	0.040
Tjp3	Q9QXY1	Tight junction protein ZO-3	7	2.12	0.043
Slc25a42	Q8R0Y8	Mitochondrial coenzyme A transporter SLC25A42	9	1.98	0.021
Mybbp1a	Q7TPV4	Myb-binding protein 1A	13	1.92	0.037
Acnat1	A2AKK5	Acyl-coenzyme A amino acid N-acyltransferase 1	4	1.81	0.046
Epb41l1	Q9Z2H5	Band 4.1-like protein 1	8	1.79	0.023
Col1a1	P11087	Collagen alpha-1(I) chain	8	1.73	0.002
Col4a2	P08122	Collagen alpha-2(IV) chain;Canstatin	5	1.69	0.006
Nomo1	Q6GQT9	Nodal modulator 1	17	1.68	0.014
Col1a2	Q01149	Collagen alpha-2(I) chain	10	1.65	0.001
Dao	P18894	D-amino-acid oxidase	11	1.63	0.002
Arpc4	P59999	Actin-related protein 2/3 complex subunit 4	2	1.62	0.007
Uqcrh	P99028	Cytochrome b-c1 complex subunit 6, mitochondrial	2	1.61	0.019
Atp5l	Q9CPQ8	ATP synthase subunit g, mitochondrial	4	1.59	0.002
Patj	Q63ZW7	Pals1-associated tight junction protein / InaD-like protein	6	1.53	0.003
Dhrs1	Q99L04	Dehydrogenase/reductase SDR family member 1	8	1.53	0.0003
Apmap	Q9D7N9	Adipocyte plasma membrane-associated protein	14	1.52	0.003
Lad1	P57016	Ladinin-1	14	1.49	0.012
Rtn4	Q99P72	Reticulon-4	8	1.49	0.012
F11r	Q88792	Junctional adhesion molecule A	2	0.70	0.050

Supplemental Table 3. Proteomic profiling of cohort 2, 8–10-week-old *Nphs2*$^{BioID2/+}$ male mice, identifies 50 enriched podocyte foot process proteins. MS analysis of *Nphs2*$^{BioID2/+}$ biotin administered mice identifies Kirrel, Tjp1, Pard3b, Ddn, and Magi2 as the top 5 proteins detected respectively, with podocin detected in the top 10. Table lists 50 significant proteins ($p \leq 0.05$) at a $\log_2$ cut off ≥ 1.49 with respective *p*-values in the far-right column. One additional immunoglobulin domain containing protein, Junction adhesion molecule 1 (Jam1 / F11r) was also identified within this MS analysis with a $\log_2$ fold change of 0.7, $p \leq 0.05$. All proteins were detected with at least two razor unique peptide sequences to the specific protein identified.

Gene Symbol	Majority Protein IDs	Protein Names	Razor + Unique Peptides	Log_2 Fold Change	*p* - Value
Kirrel	Q80W68	Kin of IRRE-like protein 1	18	5.99	0.027
Cttn	Q60598	Src substrate cortactin	14	5.70	0.034
Tjp1	P39447	Tight junction protein ZO-1	90	5.62	0.050
Pard3b	Q9CSB4	Partitioning defective 3 homolog B	38	4.21	0.050
Nebl	Q9DC07	LIM zinc-binding domain-containing Nebulette	7	3.41	0.051
Fnbp1l	Q8K012	Formin-binding protein 1-like	7	3.22	0.025
Magi2	Q9WVQ1	Membrane-associated guanylate kinase, WW and PDZ domain-containing protein 2	30	2.89	0.044
Synpo	Q8CC35	Synaptopodin	10	2.88	0.044
Ildr2	B5TVM2	Immunoglobulin-like domain-containing receptor 2	11	2.76	0.003
Tjp2	Q9Z0U1	Tight junction protein ZO-2	21	2.71	0.037
Col1a2	Q01149	Collagen alpha-2(I) chain	3	2.68	0.046
Nphs2	Q91X05	Podocin	13	2.42	0.035
Col1a1	P11087	Collagen alpha-1(I) chain	5	2.37	0.011
Ddn	Q80TS7	Dendrin	13	2.35	0.021
Pkp4	Q68FH0	Plakophilin-4	13	2.27	0.033
Cd2ap	Q9JLQ0	CD2-associated protein	3	2.20	0.034
Rdx; Msn	P26043; P26041	Radixin; Moesin	2	1.74	0.015
Afdn	Q9QZQ1	Afadin	2	1.70	0.030
Hspe1	Q64433	10 kDa heat shock protein, mitochondrial	4	1.66	0.036
Aif1l	Q9EQX4	Allograft inflammatory factor 1-like	5	1.42	0.027
Hist1h4a	P62806	Histone H4	9	1.36	0.014
Tns2	Q8CGB6	Tensin-2	3	1.30	0.038
Khk	P97328	Ketohexokinase	2	1.25	0.027
Clic1	Q9Z1Q5	Chloride intracellular channel protein 1	2	1.21	0.027
Hist2h2bb	Q8CGP2;Q8CGP1	Histone H2B type 1	6	1.04	0.049
Acadl	P51174	Long-chain specific acyl-CoA dehydrogenase, mitochondrial	7	1.01	0.025

Supplemental Table 4. Proteomic profiling of cohort 3, 8–10-week-old *Nphs2$^{BioID2/+}$* female mice, identifies 28 enriched podocyte foot process proteins. MS analysis of biotin administered female *Nphs2$^{BioID2/+}$* mice identifies Kirrel, Cttn, Tjp1, Pard3b, and Nebl as the top 5 proteins detected**.** Table lists 28 significant proteins ($p \leq 0.05$) at a log_2 cut off 1.0 with respective *p*-values in the far-right column. All proteins were detected with at least two razor unique peptide sequences to the specific protein identified.

Gene Symbol	Protein Names	*p*-Value	Log 2 Fold Change
Kirrel	Kin of IRRE-like protein 1	0.0006	7.75
Tjp1	Tight junction protein ZO-1	0.002	7.63
Nphs2	Podocin	0.019	6.70
Pard3b	Partitioning defective 3 homolog B	0.008	6.39
Magi2	Membrane Associated Guanylate Kinase, WW And PDZ Domain Containing 2	0.018	6.29
Cttn	Src substrate cortactin	0.001	5.60
Ddn	Dendrin	0.003	5.56
Nebl	LIM zinc-binding domain-containing Nebulette	0.010	4.93
Ildr2	Immunoglobulin-like domain-containing receptor 2	0.007	4.90
Fnbp1l	Formin-binding protein 1-like	0.014	4.27
Synpo	Synaptopodin	0.047	2.90
Tjp2	Tight junction protein ZO-2	0.014	4.71
Myzap	Myocardial zonula adherens protein	0.009	3.45
Cd2ap	CD2-associated protein	0.020	3.18
Col1a1	Collagen alpha-1(I) chain	0.007	2.05
Aif1l	Allograft inflammatory factor 1-like	0.014	1.86
Tns2	Tensin-2	0.022	1.75
Magi1	Membrane Associated Guanylate Kinase, WW And PDZ Domain Containing 2	0.002	5.89
Khsrp	Far upstream element-binding protein 2	0.014	4.69
Mapre1	Microtubule-associated protein RP/EB family member 1	0.019	4.56
Yes1	Tyrosine-protein kinase Yes	0.015	4.34
Tmem65	Transmembrane protein 65	0.030	4.25
Ap1g1	AP-1 complex subunit gamma-1	0.018	3.85
Erlin2	Erlin-2	0.008	3.44
Epb41l5	Band 4.1-like protein 5	0.003	3.13
S100g	Protein S100-G	0.034	3.06
Capza2	F-actin-capping protein subunit alpha-2	0.014	2.82
Atp6v1e1	V-type proton ATPase subunit E 1	0.039	2.30
Pkp4	Plakophilin-4	0.033	2.27
Canx	Calnexin	0.042	2.26
Sorbs1	Sorbin and SH3 domain-containing protein 1	0.036	2.21
Farp1	FERM, RhoGEF and pleckstrin domain-containing protein 1	0.040	2.13
Tjp3	Tight junction protein ZO-3	0.043	2.12
Mybbp1a	Myb-binding protein 1A	0.037	1.92
Cndp2	Cytosolic non-specific dipeptidase	0.041	1.83
Epb41l1	Band 4.1-like protein 1	0.023	1.79
Pls3	Plastin-3	0.029	1.76
Rdx	Radixin	0.015	1.74
Msn	Moesin	0.015	1.74
Afdn	Afadin	0.021	1.70
Col4a2	Collagen alpha-2(IV) chain;Canstatin	0.006	1.69
Nomo1	Nodal modulator 1	0.014	1.68
Col1a2	Collagen alpha-2(I) chain	0.0009	1.65
Arpc4	Actin-related protein 2/3 complex subunit 4	0.007	1.62
Sec23a	Protein transport protein Sec23A	0.030	1.59
Patj	Pals 1-associated tight junction protein / InaD-like protein	0.003	1.53
Lad1	Ladinin-1	0.012	1.49
Rtn4	Reticulon-4	0.012	1.49
Cisd2	CDGSH iron-sulfur domain-containing protein 2	0.026	1.46
Naca	Nascent polypeptide-associated complex subunit alpha	0.049	1.46
Scp2	Non-specific lipid-transfer protein	0.026	1.36
Aimp1	Endothelial monocyte-activating polypeptide 2	0.046	1.26
Dhrs7b	Dehydrogenase/reductase SDR family member 7B	0.024	1.24
Clic1	Chloride intracellular channel protein 1	0.027	1.21

Supplemental Table 5. Compiled proteomic profile of three cohorts of 8–10-week-old male and female *Nphs2$^{BioID2/+}$* mice. Table lists 54 significant proteins ($p \leq 0.05$) at a $\log_2$ cut off ≥ 1.20, with respective *p*–value listed in the far-right column. Table compiles the proteomic profiles across all three separate analyses and averages their respective $\log_2$ fold change and *p*-values. This table was subsequently utilized for *Qiagen Ingenuity Pathway Analysis* (IPA) and input into the *Database for Annotation, Visualization, and Integrated Discovery* (DAVID) for gene ontology characterization. All proteins were detected with at least 2 razor unique peptides specific to the protein denoted.

Antidody	Dilution [IF]	Dilution [WB]	Company
Goat anti - Collagen IV	[1:500]		Sigma-Aldrich, AB769, Lot 2930544
Mouse (IgG1) anti - Myozap	supernatant (straight)		Progen, clone 517.67, 651169
Rabbit anti - F11r (Jam1)	[1:50]		ABclonal, A1241, Lot 0005180101
Rabbit anti - Fnbp1l (Toca-1)	[1:100]		Bethyl Laboratories, A303-469A, Lot 1
Rabbit anti - HA	[1:500]	[1:1000]	Cell Signaling, clone C29F4, 3724S
Rabbit anti - Ildr2	[1:250]		BiCell Scientific Inc, 00303
Rabbit anti - Ildr2	[1:50]		Lifespan BioSciences LS-C205504
Rabbit anti - Ildr2	[1:50]		Invitrogen, PA5-46481, Lot WE327570
Rabbit anti - Pard3	[1:250]		Sigma-Aldrich, 07-330, Lot 2615671
Rabbit anti - podocin	[1:500]	[1:1000]	Invitrogen, PA5-79757, Lot WL3458408
Rabbit anti - WT1	[1:500]		Abcam, ab89901, LotGR3365362-7
Lotus Lectin	[1:1000]		Vector Laboratories, FL-1321, Lot ZC0914
Streptavidin - APC	[1:1000]		BD Pharmingen, 554067 Lot 7040924
Streptavidin - HRP		[1:5000]	Cell Signaling, 3999S, Lot 8

Supplemental Table 6. Antibodies. List of antibodies with respective company identifiers and concentrations for immunoflorecence (IF) and western blot (WB) analyses utilized in this investigation.

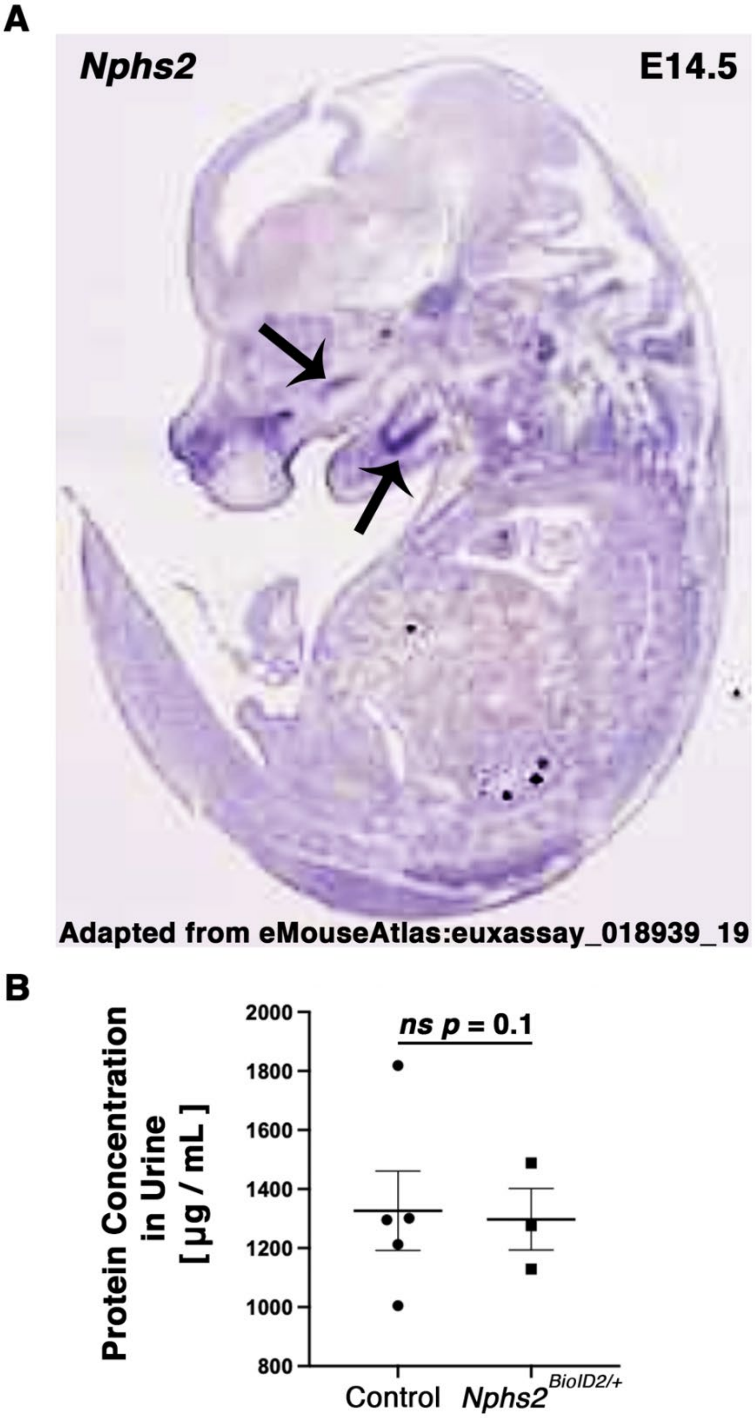

Supplemental Figure 1. ***Nphs2*** **is expressed in the developing mus** ***musculus*** **craniofacial region and** ***Nphs2$^{BioID2/+}$*** **animals do not exhibit proteinuria. (A)** *in situ* hybridization analysis of E14.5 identifies *Nphs2* transcripts within the developing craniofacial region of the mouse embryos, presumably within the region of the mandible, denoted by arrows. Image adapted from eMouseAtlas:euxassay: Image018939_19. **(B)** Proteinuria analysis of *Nphs2$^{BioID2/+}$* animals does not identify a significant increase in protein concentration within the urine, p = 0.1.

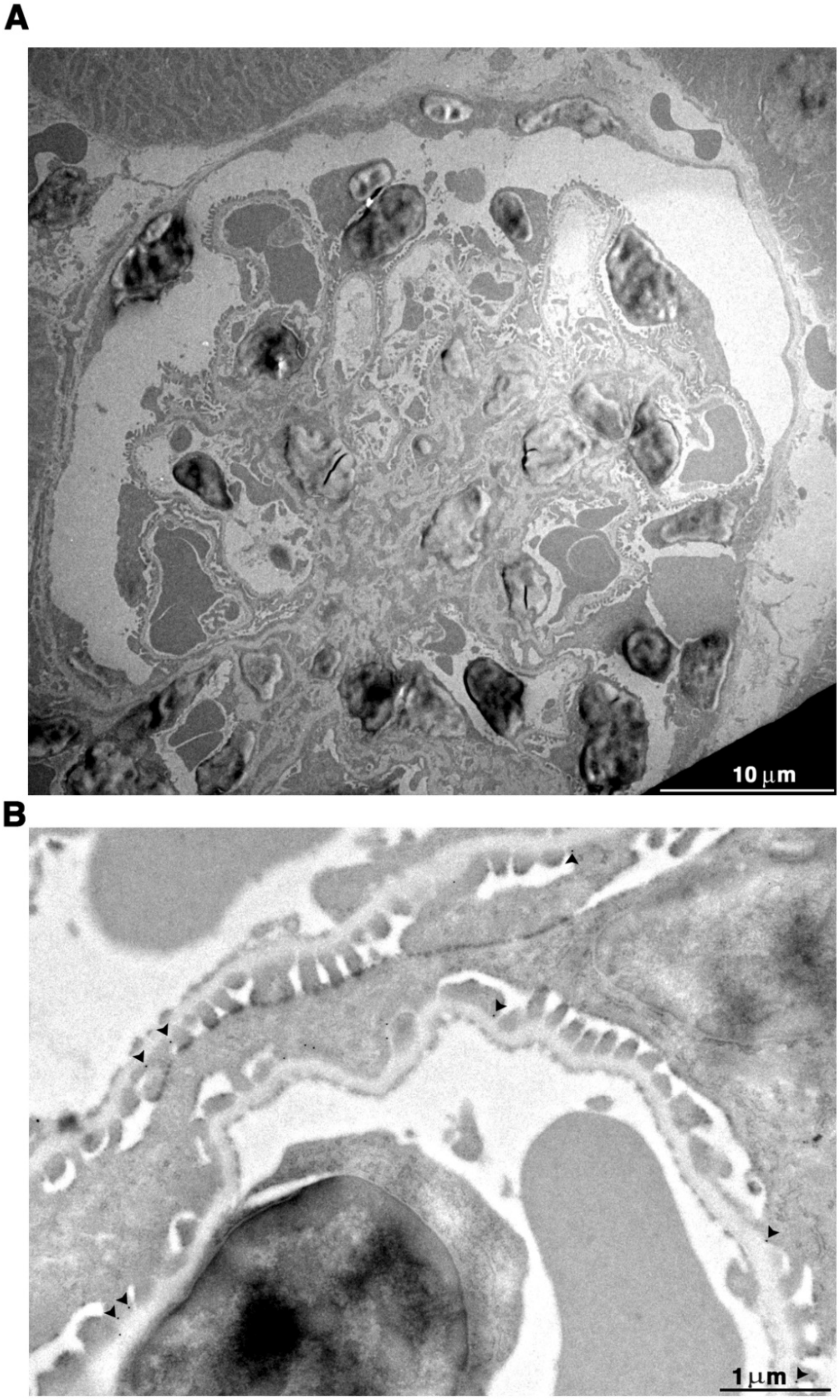

Supplemental Figure 2. *Nphs2$^{BioID2/+}$* animals evince a normal glomerular morphology by transmission electron microscopy (TEM) and display HA signal at the site of the slit diaphragm. (A) TEM cross section through a *Nphs2$^{BioID2/+}$* glomerulus shows a physiologically normal glomerular structure with a Bowman's space and podocyte foot processes wrapped around the fenestrated endothelium, Scale bar: 10 μm. **(B)** *Nphs2$^{BioID2/+}$* animals display an HA signal at the site of slit diaphragms denoted by arrowheads, with an intact contiguous glomerular basement membrane, and physiologically normal foot process structure, Scale Bar: 1 μm.

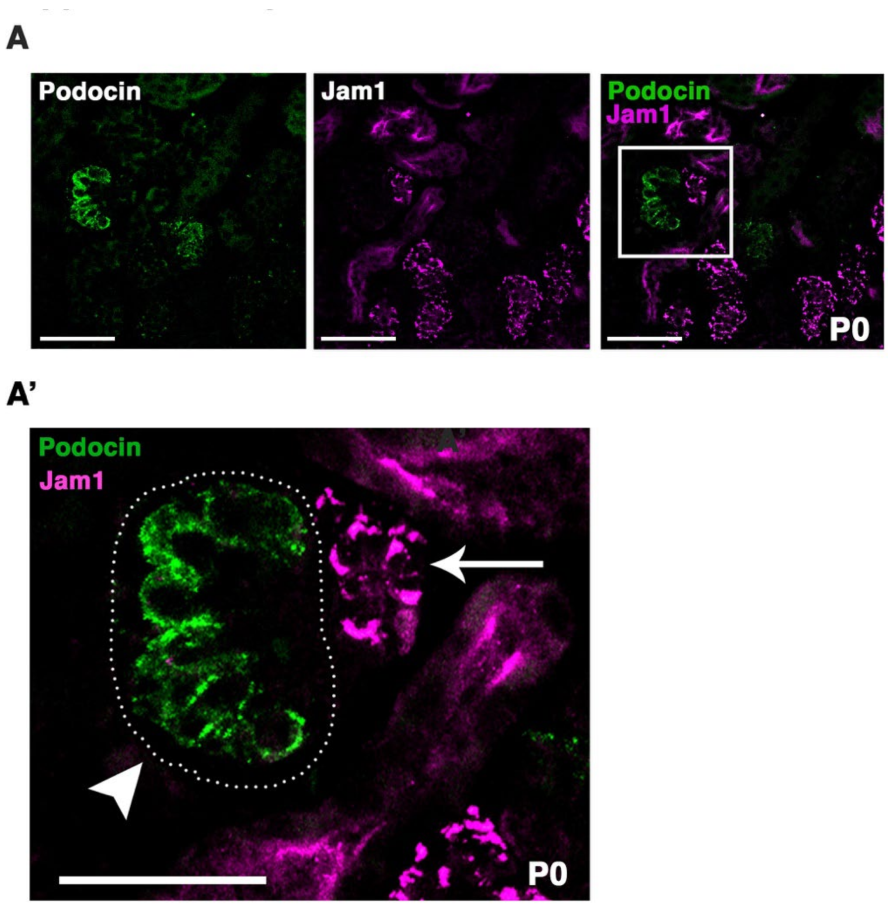

Supplemental Figure 3. Expression of immunoglobulin superfamily member Jam1/F11r in tubule cells neighboring podocytes. (A). Jam1 was identified with a 0.7 $\log_2$ fold change in a single mass spec analysis. P0 kidney sections were immunostained for Jam1 (magenta) and podocin (green). Jam1 is localized to tubule cells directly adjacent to podocytes. **(A')** Highlighted white boxed region from **(A)** is enlarged to show podocin localization restricted to the glomerulus, outlined in a dotted while circle, and Jam1 localization in neighboring tubule cells, denoted by white arrow. All scale bars: 50 μm.

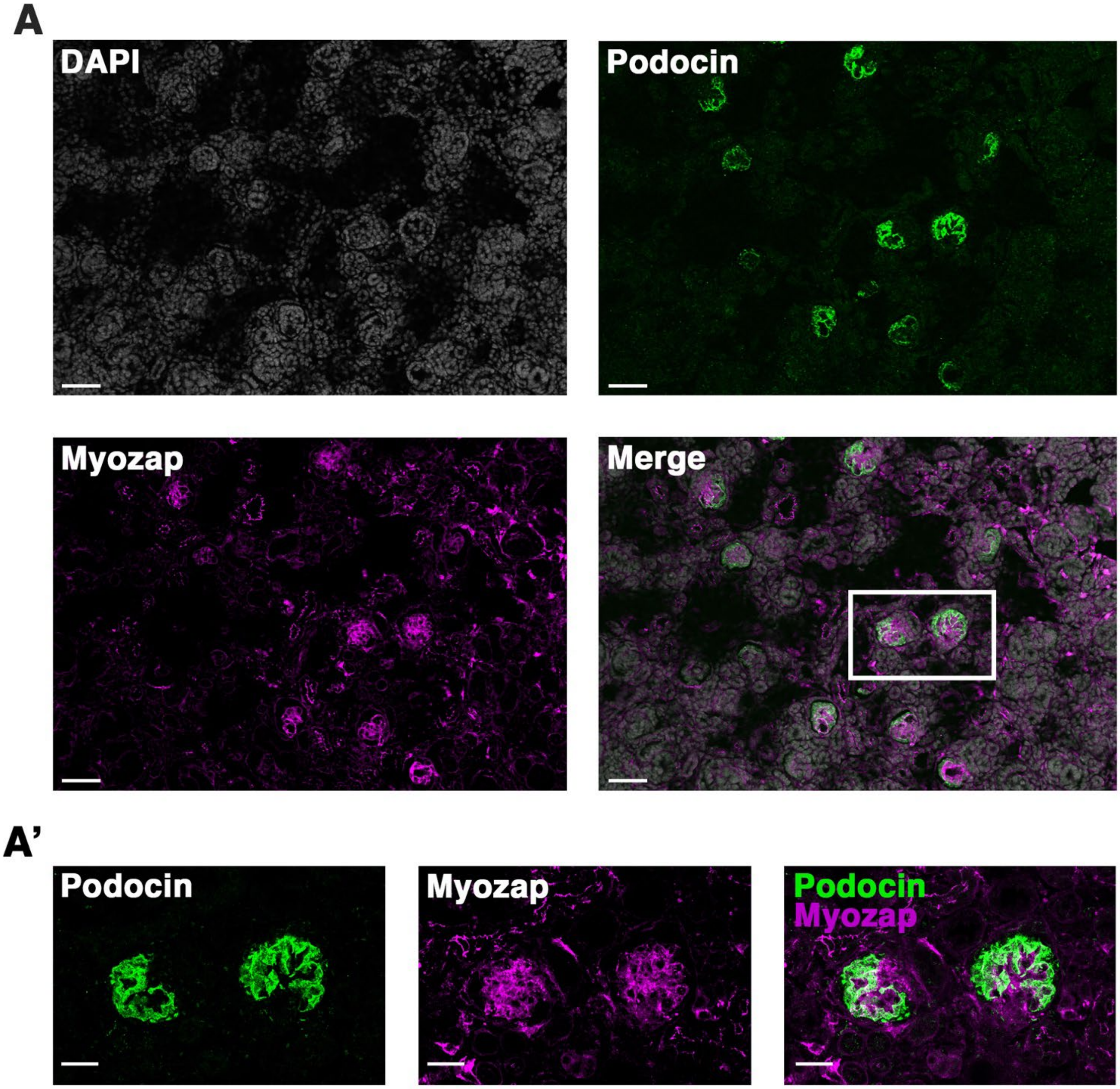

Supplemental Figure 4. Myozap localizes to endothelium of glomeruli.
(A) Immunofluorescence analysis of Myozap (magenta) identifies strong localization to the central region of the glomerulus and endothelium outside the glomerulus. Proteomic profiling detected a significant Myozap signal (3.45 Log_2 FC), in two of three MS analyses. We observe some overlap (white) between Myozap (magenta) and podocin (green), while the majority of Myozap signal does not overlap with podocin and instead localizes to presumptive endothelial cells. Scale bar: 50 μm **(A')** Highlighted white boxed region from **(A)** indicating staining of Myozap within the endothelium of the glomerulus and some overlap with podocin. Scale bar: 20 μm.

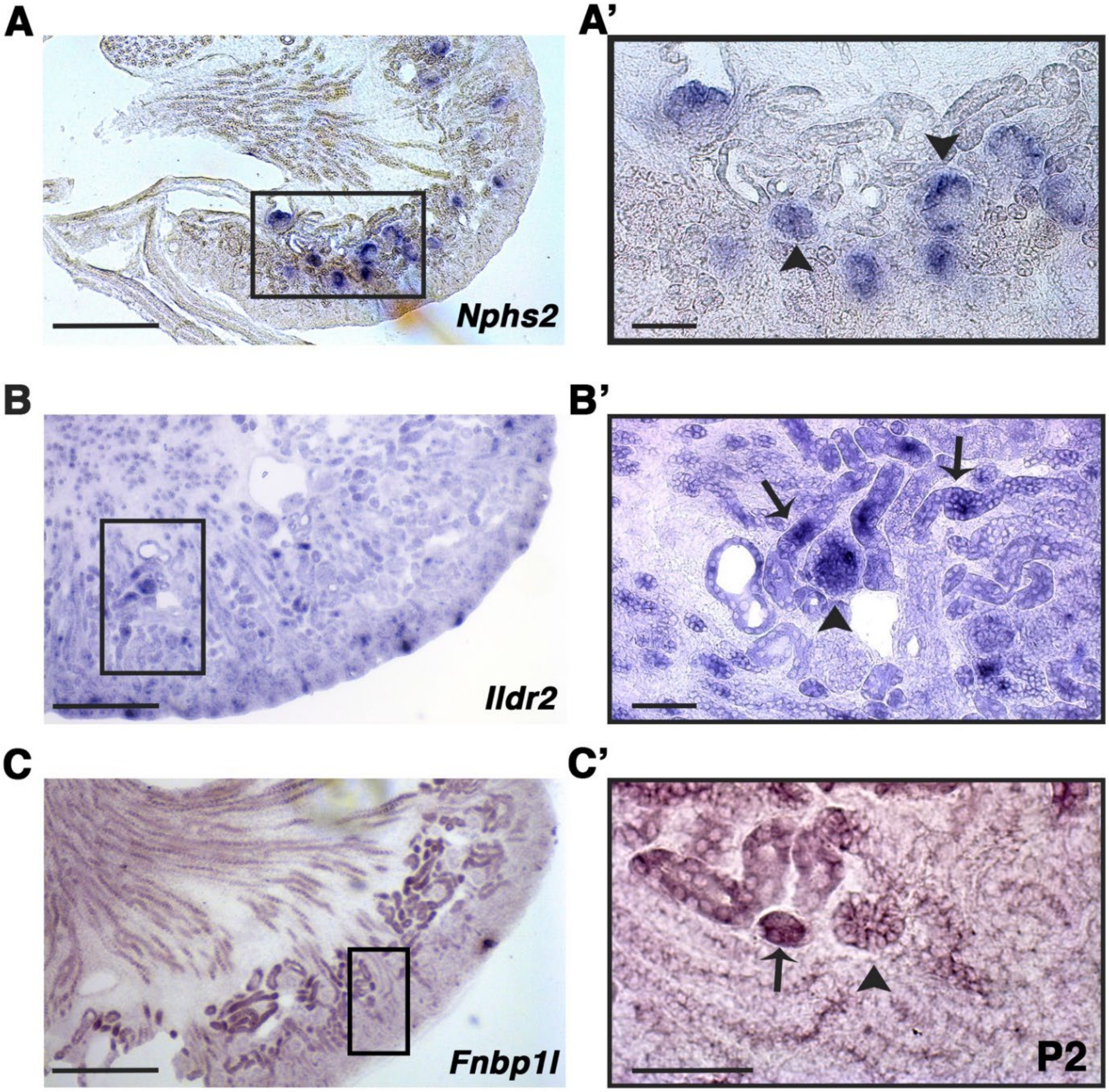

Supplemental Figure 5. *In situ* hybridization confirms glomerular and some tubule cell expression of *Ildr2* and *Fnbp1l* in P2 kidneys. P2 kidney sections hybridized with antisense riboprobes against *Nphs2* (podocin)**,** *Ildr2***,** and *Fnbp1l*. **(A)** *Nphs2* (purple) displays strong expression specifically in glomeruli at P2. The rectangular box in **(A)** is enlarged in **(A’)** to highlight expression of *Nphs2* only in glomeruli, arrowheads denote example glomeruli **(B)** *Ildr2* transcripts (purple) are identified within glomeruli and tubules. The rectangular box in **(B)** is enlarged in **(B’)** to denote expression of *Ildr2* in glomeruli, arrowhead, and in some tubules, black arrow. **(C)** *Fnbp1l* (purple) is identified in both glomeruli and tubules. The block rectangular box in **(C)** is enlarged in **(C’)** to denote expression of *Fnbp1l* in glomeruli, arrowhead, and tubules, arrow. Scale bars in A–C: 500 μm. Scale bars in A’-C’: 100 μm.

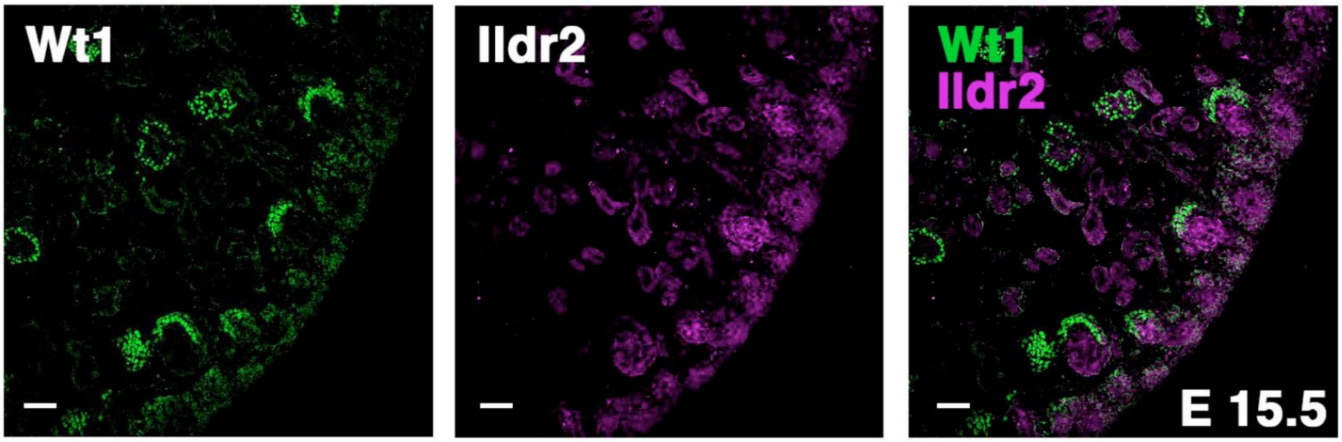

Supplemental Figure 6. Ildr2 is detected in developing nephron structures. Immunofluorescence analysis of E15.5 kidney sections co-immunostained for Ildr2 (magenta) Wt1 (podocytes, green). Scale bar: 50 μm.

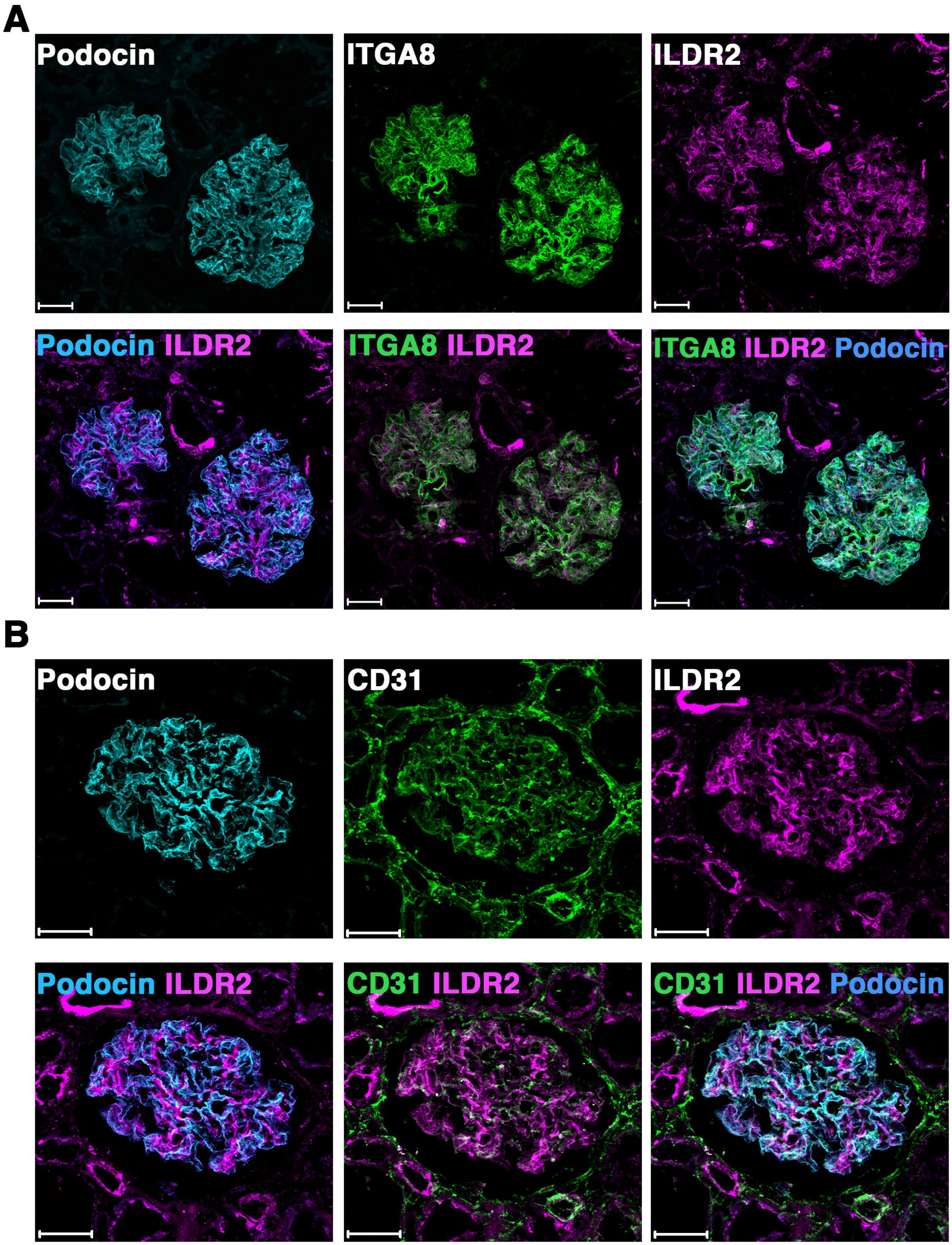

Supplemental Figure 7. ILDR2 increases in detection, specifically in podocytes, of an aged human kidney. Immunofluorescence analysis of sections from 91yo human kidney tissue identifies an increase in ILDR2 signal specifically within podocytes. **(A)** Colocalization of ITGA8 (mesangium,

green), ILDR2 (magenta), and podocin (cyan) **(B)** Colocalization of CD31 (endothelium, green), ILDR2 (magenta), and podocin (cyan). Scale bar: 50 μm.
